# Cross-Talk between cAMP and MAPK Pathways in HSD11B2 Induction by hCG in Placental Trophoblasts

**DOI:** 10.1371/journal.pone.0107938

**Published:** 2014-09-17

**Authors:** Qun Shu, Wenjiao Li, Jianneng Li, Wangsheng Wang, Chao Liu, Kang Sun

**Affiliations:** 1 Shanghai Key Laboratory for Assisted Reproduction and Reproductive Genetics, Center for Reproductive Medicine, Renji Hospital, School of Medicine, Shanghai Jiao Tong University, Shanghai, China; 2 Changning Maternity and Infant Health Hospital, Shanghai, China; 3 School of Life Sciences, Fudan University, Shanghai, China; University of Oslo, Norway

## Abstract

Overexposure of the fetus to glucocorticoids in gestation is detrimental to fetal development. The passage of maternal glucocorticoids into the fetal circulation is governed by 11beta-Hydroxysteroid Dehydrogenase Type 2 (HSD11B2) in the placental syncytiotrophoblasts. Human chorionic gonadotropin (hCG) plays an important role in maintaining placental HSD11B2 expression via activation of the cAMP pathway. In this study, we investigated the relationship between the activation of the cAMP pathway by hCG and subsequent phosphorylation of extracellular signal-regulated kinase1/2 (ERK1/2) or p38 mitogen-activated protein kinase (MAPK) pathways in the regulation of placental HSD11B2 expression in human placental syncytiotrophoblasts. We found that treatment of the placental syncytiotrophoblasts with either hCG or dibutyl cAMP (dbcAMP) could promote the phosphorylation of p38 and ERK1/2. Inhibition of p38 MAPK with SB203580 not only reduced the basal HSD11B2 mRNA and protein levels but also attenuated HSD11B2 levels induced by either hCG or dbcAMP. By contrast, inhibition of ERK1/2 with PD98059 increased the basal mRNA and protein levels of HSD11B2 and had no effect on HSD11B2 mRNA and protein levels induced by either hCG or dbcAMP. These data suggest that p38 MAPK is involved in both basal and hCG/cAMP-induced expression of HSD11B2, and ERK1/2 may play a role opposite to p38 MAPK at least in the basal expression of HSD11B2 in human placental syncytiotrophoblasts and that there is complicated cross-talk between hCG/cAMP and MAPK cascades in the regulation of placental HSD11B2 expression.

## Introduction

Appropriate amounts of glucocorticoids are crucial for the normal development and maturation of the fetus [1]. However inappropriate exposure of the fetus to glucocorticoids during development may not only cause intrauterine growth retardation but also program the development of cardiovascular diseases, diabetes and cognitive disorders in later life [2], [3]. Despite the adverse effects of excessive glucocorticoids in fetal development, maternal adrenal glands undergo progressive hyperplasia and secrete increasing amounts of glucocorticoids throughout gestation [4]. By contrast, the fetal adrenal glands are mainly specialized in the production of dehydroepiandrosterone, the precursor for estrogen synthesis in the placenta [5]. Although the production of glucocorticoids by the fetal adrenal glands also tends to increase at late gestation, the maternal plasma concentration of cortisol is 5–10 times higher than that of the fetal plasma [6]. To finely tune the passage of maternal glucocorticoids which are highly lipid-permeable steroids, into the fetal circulation, there exists a glucocorticoid inactivating enzyme, i.e. 11beta-Hydroxysteroid Dehydrogenase Type 2 (HSD11B2) [7]–[9] which converts biologically active cortisol in maternal circulation into inactive cortisone, in the syncytial layer of the placental villi [10], [11]. Thus understanding of the regulation of placental HSD11B2 expression may represent a key component in the overall understanding of the molecular mechanisms that govern fetal development.

In addition to the expression of HSD11B2, the placental syncytial layer also synthesizes and secrets a large amount of human chorionic gonadotropin (hCG) [12]–[14] from early pregnancy. Human chorionic gonadotropin shares the same receptor with pituitary luteinizing hormone (LH). The hCG/LH receptor is coupled with Gαs protein and uses the cyclic adenosine monophosphate (cAMP) as a second messenger for signal transduction [15]. The role of hCG for the maintenance of pregnancy is best known for maintaining progesterone synthesis, regulating implantation, promoting uterine quiescence and controlling hormone secretion from the trophoblast [14], [16]–[18]). In addition, we have demonstrated that hCG plays an important role in maintaining placental HSD11B2 expression via activation of the cAMP pathway [19], [20]. The promoter region of HSD11B2 is rich in CpG islands within which harbor multiple binding sites for the transcription factor SP1 [21], [22]. We have demonstrated that activation of the cAMP pathway increases the enrichment of SP1 at the promoter of HSD11B2 thereby leading to increased expression of HSD11B2 in human placental syncytiotrophoblasts [23].

Cross-talk between cAMP and MAPK pathways has been documented at multiple levels in a number of cell types [24]–[26]. Extracellular signal-regulated kinase1/2 (ERK1/2), p38 mitogen-activated protein kinase (p38 MAPK) and c-Jun N-terminal kinase (JNK) are the key kinases of MAPK pathways. Activation of the cAMP pathway was reported to be associated with the phosphorylation of ERK1/2, p38 and JNK [27]–[29]. Of interest, the p38 MAPK and ERK1/2 MAPK were shown to be a positive and negative regulator of placental HSD11B2 respectively, but JNK MAPK was found to have no effect [30], [31]. However, the relationship between the activation of the cAMP pathway by hCG and subsequent phosphorylation of ERK1/2 or p38 in the regulation of placental HSD11B2 expression remains largely unknown. Therefore, the present study was designed to address this issue using our established primary human trophoblast cells as an *in vitro* model system.

## Materials and Methods

### Human placental trophoblast cell culture

Human placentae were obtained from uncomplicated normal and term (38–40 wk) pregnancies after elective cesarean section without labor. Since the placentae were usually discarded after delivery, oral informed consent of using these placentae for this study was obtained from patients and the consent information was recorded in the study record book. Both the consent procedure and the study protocol were approved by the Ethics Committee of School of Life Sciences, Fudan University. Placental trophoblast cells were prepared using a modified Kliman’s method [32] as described previously [33]. In brief, tissue aliquots were removed randomly from the maternal side of the placenta and digested with 0.125% trypsin (Sigma Chemical Co., St. Louis, MO) in DMEM (Life Technologies, Inc., Grand Island, NY). The placental cytotrophoblasts were purified using a 5–75% Percoll (Sigma) gradient at step increments of 5%. The cytotrophoblasts were plated at a density of 1.5×10^6^ cells per well in six-well plates for culture at 37°C 5% CO2, 95% air in DMEM containing 10% newborn calf serum (NCS) (Life Technologies) to allow syncytialization *in vitro* for 3 days. The culture medium was replaced with serum-free medium 3 days after plating and the cells were then treated with dbcAMP, an analog of cAMP (Sigma) or hCG (Sigma), for 24 h in the presence and absence of p38 MAPK inhibitor SB203580 (Sigma) or ERK1/2 inhibitor PD98059 (Sigma). The concentrations of these reagents are shown in the Results section and in the corresponding figure legends. Total RNA and cell proteins were then extracted for the measurements of HSD11B2 and SP1 mRNA and protein levels with quantitative real-time PCR (qRT-PCR) and Western blotting. The time course of p38 and ERK1/2 phosphorylation by treatment of the syncytiotrophoblasts with dbcAMP or hCG for 0, 15, 30, and 60 min was studied. Total cell protein was then extracted in the presence of phosphatase inhibitor (Active Motif, Carlsbad,CA). Phosphorylated and total p38 and ERK1/2 MAPK protein levels were analyzed with Western blotting. In order to assess the cell death and syncytialization caused by dbcAMP and hCG treatment, lactate dehydrogenase (LDH) level in the culture medium was measured with LDH Cytotoxicity Assay Kit (Cayman, Ann Arbor, MI) and the E-cadherin level was examined with Western blotting after treatment.

### Extraction of RNA and measurement of HSD11B2 and SP1 mRNA levels with qRT-PCR

Total RNA was extracted from the placental syncytiotrophoblasts 24 h after treatment using an UNIQ-10 RNA extraction kit (Sangon Biotech, Shanghai, China). After determination of RNA concentration, mRNA was reverse transcribed to cDNA with oligo (dT) 12–18 primer using Moloney murine leukemia virus reverse transcriptase (Promega) and cDNA was utilized for subsequent measurement of HSD11B2 and SP1 mRNA levels with qRT-PCR using power SYBR green PCR master mix (Toyobo, Osaka, Japan). The annealing temperature was set at 61°C. The absolute mRNA levels in each sample were calculated according to a standard curve set up using serial dilutions of known amounts of specific PCR product templates against corresponding cycle threshold values. To control for sampling errors, qRT-PCR for the housekeeping gene ACTB was performed on each sample. The ratio of the copy numbers of target gene over ACTB in each sample was obtained to normalize the expression of the target gene. The primer sequences for amplifying human HSD11B2, SP1 and ACTB genes are given in Table 1.

**Table 1:** Primer sequences used for PCR.

Genes	Primer sequences (5′–3′)	Genbank accession no.	Products (bp)
*HSD11B2*	GACATGCCATATCCGTGCTT(F)	NM_000196	118
	GCTGGATGATGCTGACCTTG(R)		
*SP1*	GTTTCCTTGGGGCAGACCAG(F)	NM_138473	288
	TCCTTCCTCTCCACCTGCTG(R)		
ACTB	GGGAAATCGTGCGTGACATTAAG(F)	NM_001101	275
	TGTGTTGGCGTACAGGTCTTTG(R)		

### Extraction of protein and analysis with Western blotting

Total cell protein was extracted from human placental syncytiotrophoblasts using an extraction kit from Active Motif (Carlsbad,CA). The protein levels of HSD11B2, SP1, E-cadherin, p38 and ERK1/2 were examined following a standard Western blotting protocol. Briefly, 50 µg protein of each sample were electrophoresed in 10% SDS-polyacrylamide gel and transferred to the nitrocellulose blot. After blocking, the blot was incubated with 1∶5000 dilution of HSD11B2 antibody (Santa Cruz) or 1∶500 dilution of SP1 antibody (Santa Cruz Biotechnology, Santa Cruz, CA, USA) or 1∶500 dilution of E-cadherin antibody (Santa Cruz) or 1∶500 dilution of antibodies recognizing phosphorylated p38 (Santa Cruz) and total p38 (Santa Cruz) or 1∶200 dilution of antibodies recognizing phosphorylated ERK1/2 (Santa Cruz) and total ERK1/2 (Santa Cruz) overnight. After washing, the blot was incubated with appropriate secondary antibody conjugated with horseradish peroxidase (Santa Cruz) for 1 h. The enhanced chemiluminescent detection system (Amersham) was used to detect the bands with peroxidase activity. The same blot was reprobed for β-actin for loading control. The level of phosphorylated p38 is expressed as the ratio of band densities of phosphorylated p38 and ERK1/2 over total p38 and ERK1/2.

### Statistical analysis

All data are reported as mean ± S.E.M. of repeated experiments on the placental syncytiotrophoblasts prepared from different placentae. After examination of normal distribution, paired Student’s t-test or one-way ANOVA test followed by the Student–Newman–Keuls test was used where appropriate to assess significant differences. Significance was set at P<0.05.

## Results

### Effects of dbcAMP and hCG on the phosphorylation of p38 and ERK1/2 MAPKs in human placental syncytiotrophoblasts

**Figure 1:**
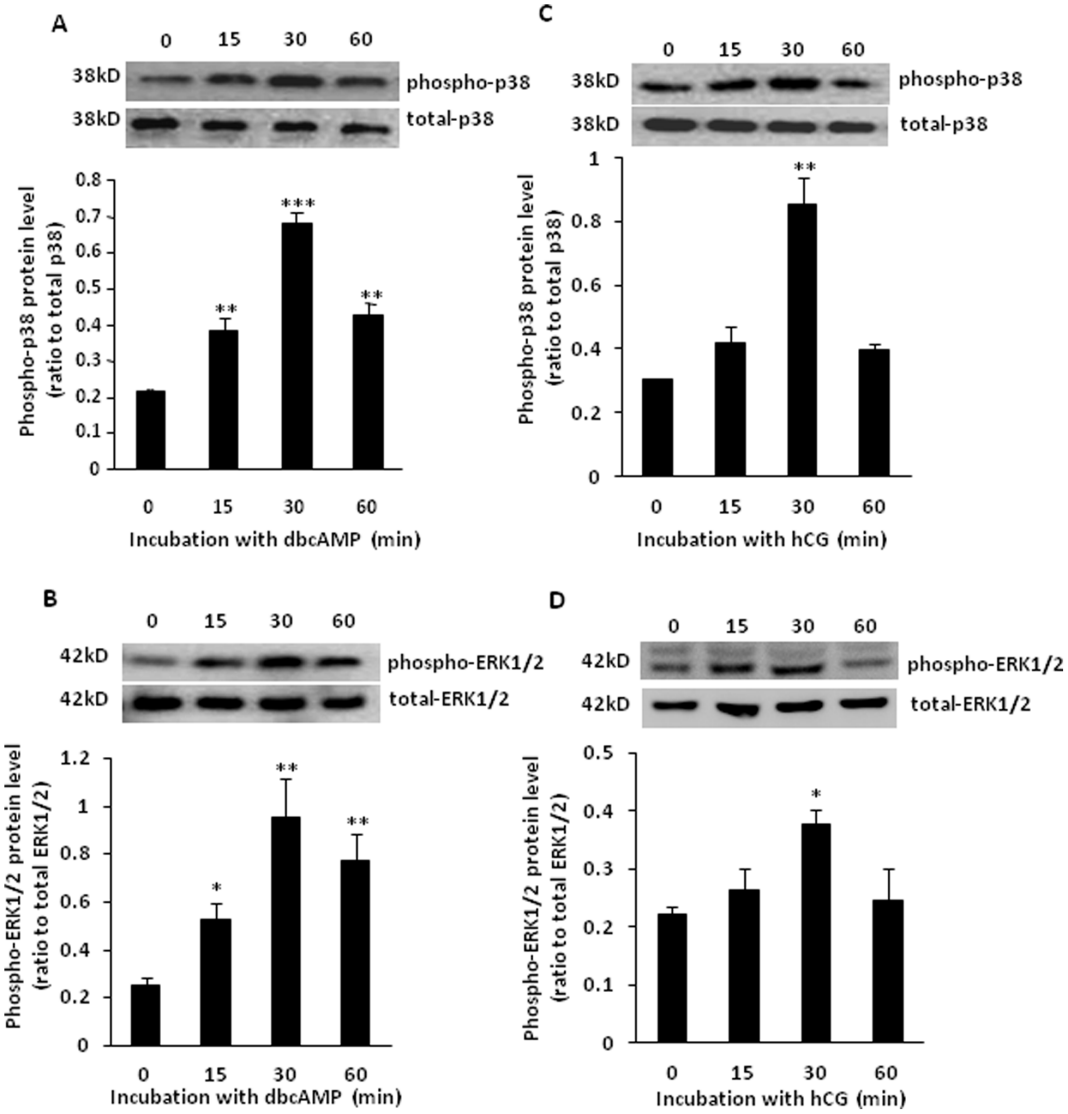
Phosphorylation of p38 MAPK (P-p38) and ERK1/2 induced by dbcAMP (100 µM) and hCG (10 IU/ml) in human placental syncytiotrophoblasts. Upper panels of each bar graph are the representative blots. The bar graphs are the average data of three experiments. *P<0.05, **P<0.01,***P<0.001 versus 0 min.

Treatment of the syncytiotrophoblasts with dibutyl cyclic AMP (dbcAMP, 100 µM; 0, 15, 30, and 60 min) significantly increased the phosphorylation of p38 and ERK1/2 MAPKs in a time-dependant manner with the maximal effect observed at 30 min (Fig. 1A and Fig. 1B). Treatment of the syncytiotrophoblasts with hCG (10 IU/ml) significantly increased the phosphorylation of p38 and ERK1/2 at 30 min, but not at other time points used in this study (Fig. 1C and Fig. 1D). Treatment with either dbcAMP or hCG did not affect LDH level in the culture medium and cellular E-cadherin level (data not shown), suggesting the treatments did not change the cell viability and cause further syncytialization.

### Role of p38 MAPK in the induction of HSD11B2 and SP1 by hCG/dbcAMP in human placental syncytiotrophoblasts

**Figure 2:**
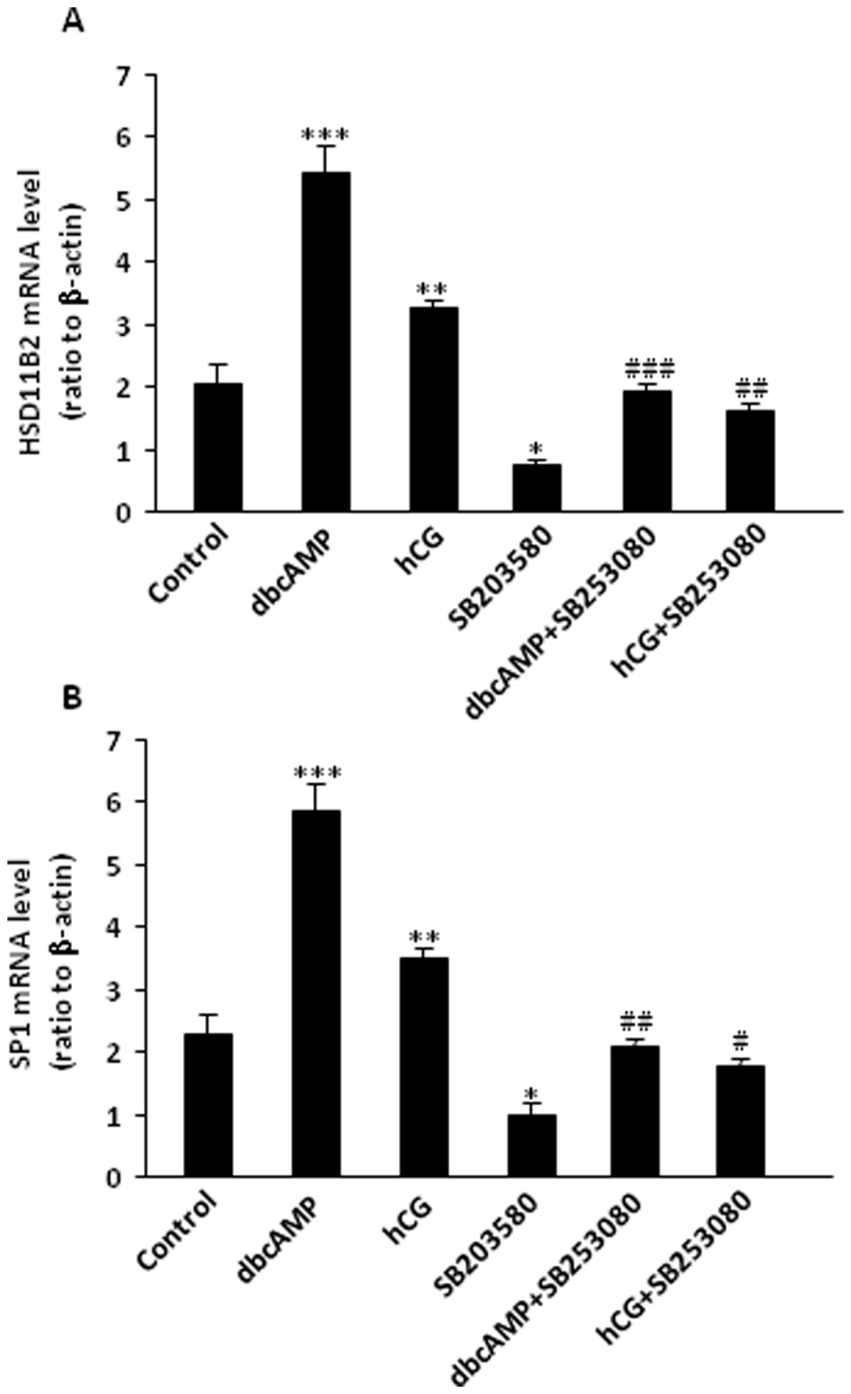
(A) Effect of SB203580 (p38 MAPK inhibitor, 10 µM) on the basal and dbcAMP (100 µM) and hCG (10 IU/ml)-induced HSD11B2 mRNA expression in human placental syncytiotrophoblasts. *p<0.05, **p<0.01, ***p<0.001 versus control; ## p<0.01 ### p<0.001 versus treatment with db-cAMP and hCG n = 4; (B) Effect of SB203580 (p38 MAPK inhibitor, 10 µM) on the basal and dbcAMP (db cAMP, 100 µM) and hCG (10 IU/ml)-induced SP1 mRNA expression in human placental syncytiotrophoblasts. *p<0.05, **p<0.01, ***p<0.001 versus control;# p<0.05, ## p<0.01 versus treatment with dbcAMP and hCG (n = 4).

**Figure 3:**
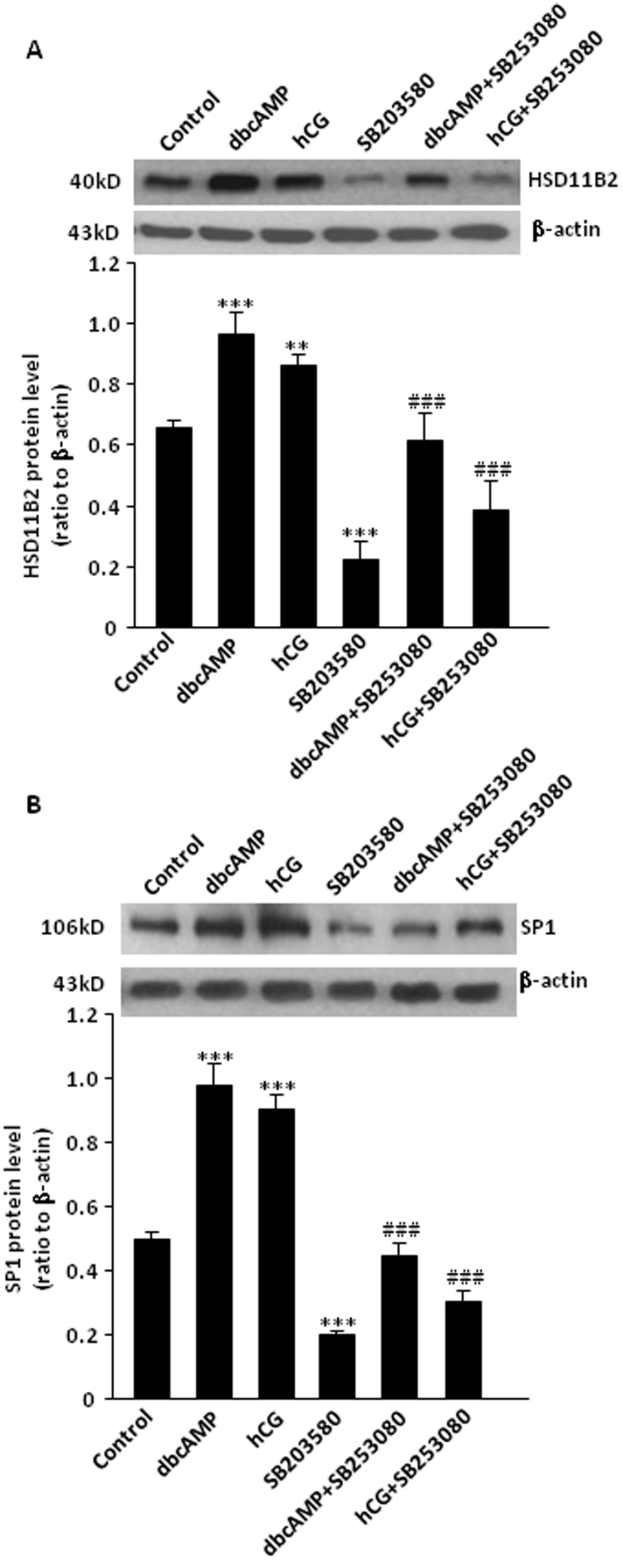
(A) Effect of SB203580 (p38 MAPK inhibitor, 10 µM) on the basal and dbcAMP (100 µM) and hCG (10 IU/ml)-induced HSD11B2 protein level in human placental syncytiotrophoblasts. *p<0.05, **p<0.01, ***p<0.001 versus control; ## p<0.01 ### p<0.001 versus treatment with dbcAMP and hCG, n = 4. (B) Effect of SB203580 (p38 MAPK inhibitor, 10 µM) on the basal and dbcAMP, 100 µM) and hCG (10 IU/ml)-induced SP1 protein level in human placental syncytiotrophoblasts. ***p<0.001 vs control; ### p<0.001 versus treatment with dbcAMP and hCG (n = 4).

SB203580 (10 µM), an inhibitor of p38 MAPK, decreased not only the basal levels of HSD11B2, SP1 mRNA (Fig. 2A and Fig. 2B) and protein (Fig. 3A and Fig. 3B), but also the levels of HSD11B2, SP1 mRNA and protein induced by either hCG (10 IU/ml) or dbcAMP (100 µM) significantly (Fig. 2 and Fig. 3), suggesting that the induction of HSD11B2 and SP1 expression by hCG/cAMP is, at least in part, mediated via p38 MAPK in human placental syncytiotrophoblasts.

### Role of ERK1/2 MAPK in the induction of HSD11B2 and SP1 by hCG/dbcAMP in human placental syncytiotrophoblasts

**Figure 4:**
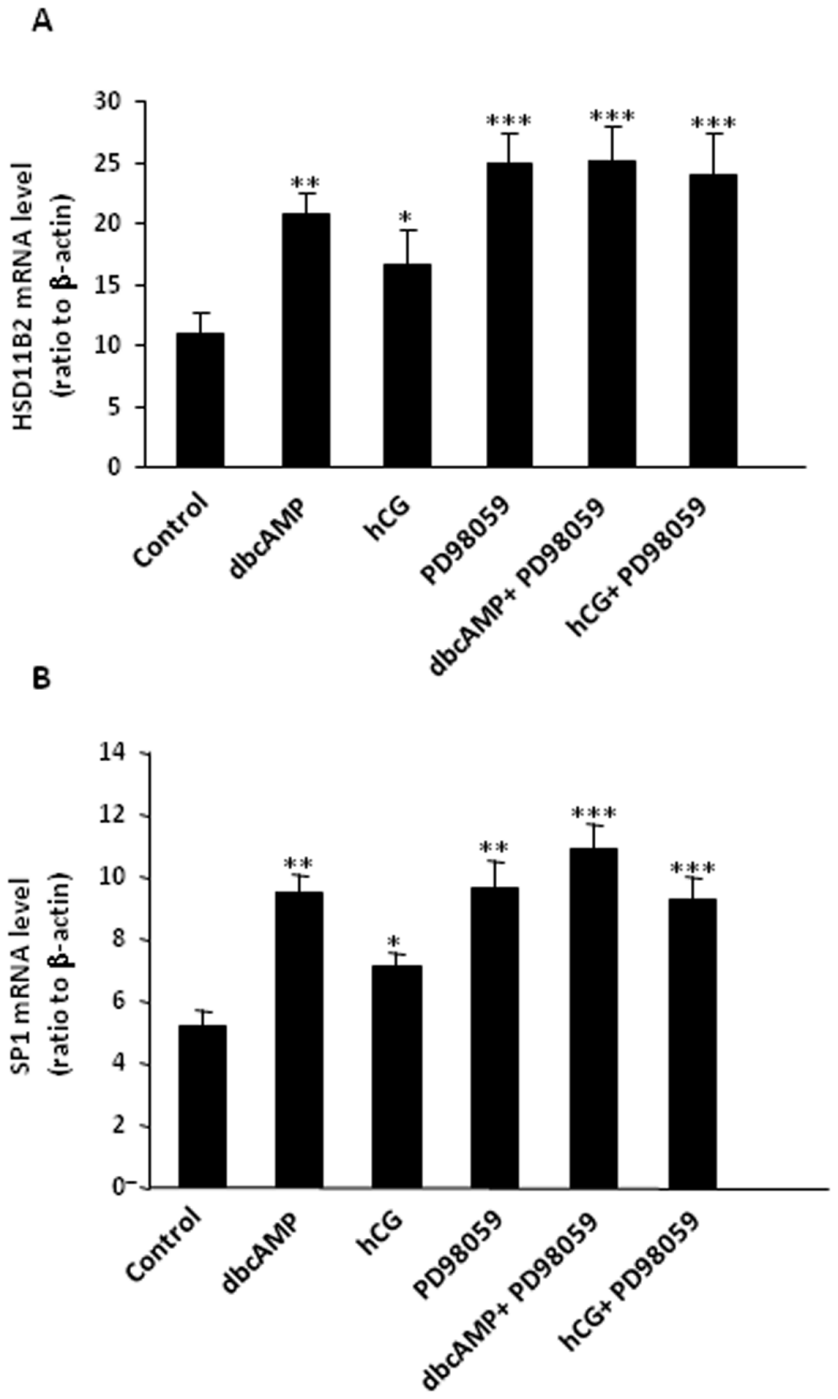
(A) Effect of PD98059(ERK1/2 MAPK inhibitor, 50 µM) on the basal and dbcAMP (100 µM) and hCG (10 IU/ml)-induced HSD11B2 mRNA expression in human placental syncytiotrophoblasts. *p<0.05, **p<0.01, ***p<0.001 versus control (n = 4) (B) Effect of PD98059 (ERK1/2 MAPK inhibitor, 50 µM) on the basal and dbcAMP(100 µM) and hCG (10 IU/ml)-induced SP1 mRNA expression in human placental syncytiotrophoblasts. *p<0.05, **p<0.01, ***p<0.001 versus control (n = 4).

**Figure 5:**
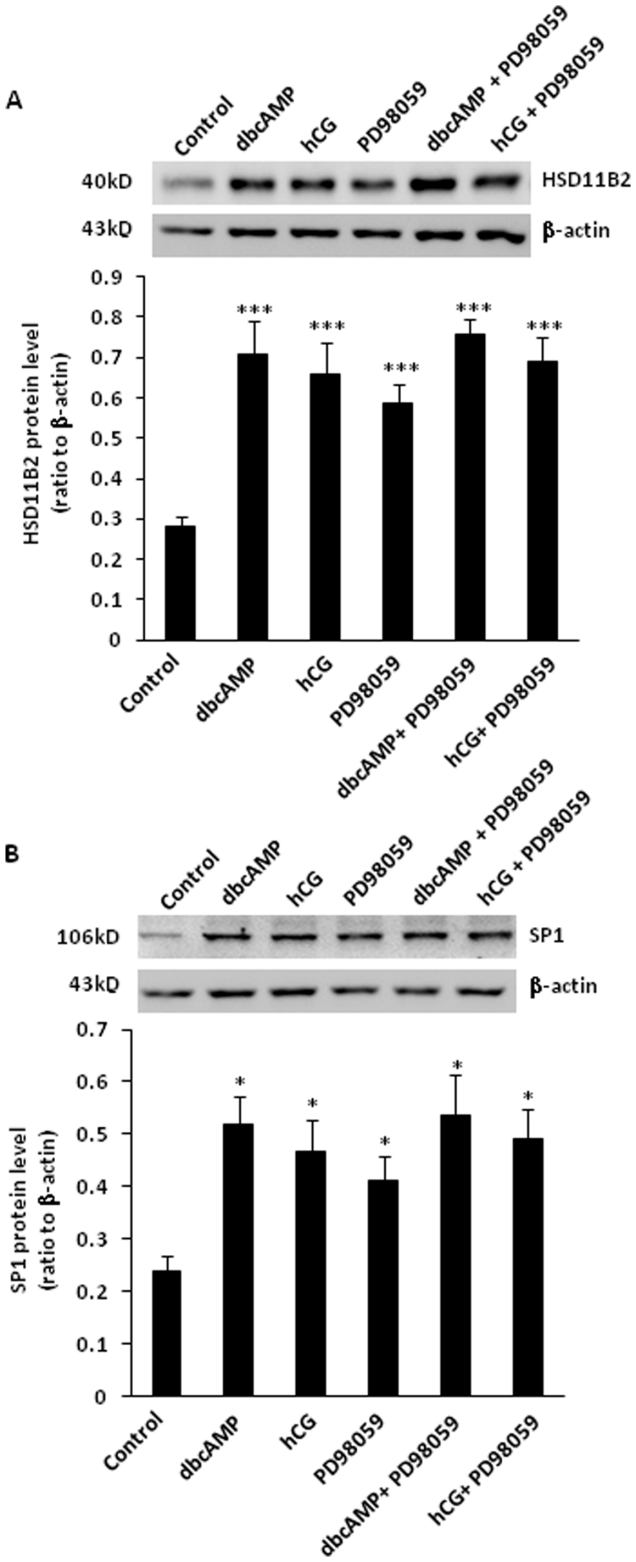
(A) Effect of SB203580 (p38 MAPK inhibitor, 10 µM) on the basal and dbcAMP (100 µM) and hCG (10 IU/ml)-induced HSD11B2 protein level in human placental syncytiotrophoblasts. *p<0.05 versus control(n = 4). (B) Effect of SB203580 (p38 MAPK inhibitor, 10 µM) on the basal and dbcAMP (100 µM) and hCG (10 IU/ml)-induced SP1 protein level in human placental syncytiotrophoblasts. ***p<0.001 versus control (n = 4).

PD98059 (50 µM), an inhibitor of ERK1/2 MAPK, increased the basal levels of HSD11B2, SP1 mRNA (Fig. 4A and Fig. 4B) and protein (Fig. 5A and Fig. 5B), but had no effect on the levels of HSD11B2 and SP1 mRNA protein induced by either hCG (10 IU/ml) or dbcAMP (100 µM) (Figs. 4 and 5).

## Discussion

The placental barrier is not merely a physical structure that separates the maternal and fetal circulations, but also a molecular fence that prevents unwanted maternal materials entering the fetal environment, and *vice versa*. Expression of HSD11B2 enzyme in the placental syncytiotrophoblasts forms such a molecular barrier for the protection of the fetus from excessive glucocorticoids of maternal origin [7]–[9], [34]. Accumulating evidence indicates that high concentration of glucocorticoids is a risk factor for low birth weight and developmental origin of diseases [2], [3]. It has been reported that HSD11B2 deficiency caused by point mutation is associated with intrauterine growth retardation (IUGR) in human pregnancies [35] and that HSD11B2^−/−^ mice exhibit IUGR phenotype [36]. Appropriate expression level of HSD11B2 in the placenta is thus of pivotal importance for the normal development of the fetus. As such, continuous efforts have been directed to the understanding of the regulation of HSD11B2 in human placenta. Studies have shown that estrogen [37], hCG [20] and glucocorticoids [38] can increase HSD11B2 expression, while prostaglandins [39], progesterone [19], hypoxia [40] and cadmium [41] can decrease HSD11B2 expression in human placenta. Of interest, IUGR is characterized by a significant decrease in hCG concentration in the circulation, and this has previously been used as an indicator of IUGR [42]. Our previous study has demonstrated that hCG plays an important role in maintaining HSD11B2 expression in human placenta. In this study we demonstrated that activation of hCG/cAMP pathway phosphorylated both ERK1/2 and p38 MAPKs in human placental syncytiotrophoblasts. Activation of p38 pathway might account for maintaining the basal as well as hCG/dbcAMP-induced expression of HSD11B2, whereas ERK1/2 MAPK might play a role opposite to p38 MAPK in the basal expression of HSD11B2, and moreover ERK1/2 MAPK appears not to be associated with the regulation of HSD11B2 by hCG/dbcAMP despite the observation that the phosphorylation of ERK1/2 MAPK was increased by hCG/dbcAMP. Alternatively the cells may also be maximally stimulated by inhibition of ERK1/2 MAPK at the basal state in terms of HSD11B2 expression and could not be further stimulated by exogenous hCG/dbcAMP.

It is well known that hCG is one of the earliest hormone secreted by the placenta and there is sophisticated cross-talk between the cAMP and MAPK pathways [24]–[27]. As an example, the regulation of leptin production by hCG is the result of cross-talk between hCG/cAMP pathway and p38 as well as ERK1/2 pathways in human placenta [28], [43]. Here we demonstrate that treatment of placental syncytiotrophoblasts with either hCG or dbcAMP increase the phosphorylation of p38 and ERK1/2 MAPKs, which provides further evidence for the cross-talk between the canonical hCG/cAMP and MAPK pathways.

MAPK pathways are essential for invasion [44], proliferation [45], differentiation [46] and apoptosis [47] in trophoblast cells. It is interesting that opposing effects of ERK1/2 and p38 on cell apoptosis were reported [48]. In this study we found that dbcAMP or hCG activate both ERK1/2 and p38 but did not affect the trophoblast viability suggesting the effects observed were unlikely due to the cell death caused by the treatment. Recent studies have shown that, of the three major MAPKs, p38 and ERK1/2 are positive and negative regulators of placental HSD11B2 respectively while JNK does not appears to be involved in the regulation of placental HSD11B2 [30], [31]. Here we confirm that p38 MAPK maintains basal placental HSD11B2 expression and ERK1/2 MAPK down-regulates basal placental HSD11B2 expression. Moreover, we provide evidence that hCG up-regulates HSD11B2 in human trophoblastic cells via the cross-talk between the cAMP and p38 MAPK pathways, while ERK1/2 MAPK appears to have either opposite or no effect on the stimulated expression of HSD11B2 by the hCG/cAMP pathway. These results illustrate that there is complicated cross-talk between hCG/cAMP and MAPK cascades in the regulation of placental HSD11B2 expression. Further studies to identify the upstream factors that activate p38 and ERK1/2 MAPK signaling pathways in the regulation of placental HSD11B2 expression may lead to the findings of interactions between interactions between hormones and effectors that would equilibrate the HSD11B2 expression in human placenta. Likewise, the diversity of effects mediated by p38 and ERK1/2 pathways was also reported in other cell context[48]–[53]. For instance, suppression of p38 activation by SB203580 reduced the extent of apoptosis of the HeLa cells, while inhibition of ERK1/2 with PD98059 increased apoptosis [50], suggesting that balanced roles of p38 and ERK1/2 MAPKs may exist in apoptosis. Coordinated operation between these signaling cascades is also exemplified in pain-associated spatial and temporal plasticity in the hippocampus [53]. Inhibition of the ERK1/2-mediated signaling pathway significantly decreased pain-enhanced long-term potentiation (LTP) whereas blockade of p38 MAPK pathway dramatically increased the potentiation [53].

Previous studies have demonstrated that SP1 regulates both basal and cAMP pathway-induced expression of HSD11B2 in human placenta [23]. Here we demonstrate that there are parallel changes of SP1 and HSD11B2 at both mRNA and protein levels following treatment with hCG/cAMP and manipulation of both p38 and ERK1/2 MAPKs, which provides further evidence for the involvement of SP1 in HSD11B2 expression in human placental syncytiotrophoblasts.

In conclusion, our present data demonstrate that p38 MAPK maintains basal placental HSD11B2 expression and ERK1/2 MAPK down-regulates basal placental HSD11B2 expression. Moreover, p38 MAPK is involved in the induction of HSD11B2 expression by hCG/cAMP pathway, and ERK1/2 may play a role opposite to p38 MAPK at least in the basal expression of HSD11B2 in human placental syncytiotrophoblasts, and there is complicated cross-talk between hCG/cAMP and MAPK cascades in the regulation of placental HSD11B2 expression.
